# Enhancing competitiveness in accommodation management: sustainability-led strategies for tourism and event destinations

**DOI:** 10.3389/fspor.2026.1802932

**Published:** 2026-04-13

**Authors:** Toyirova Sarvinoz Atoevna, Odil Radjabov, Istamkhuja Olimovich Davronov, Kamol Yuldashev

**Affiliations:** Department of Tourism and Hotel Management, Bukhara State University, Bukhara, Uzbekistan

**Keywords:** accommodation sector, Central Asia, competitiveness, destination branding, event tourism, green certification, infrastructure, quality standards

## Introduction

The global tourism industry has experienced significant structural changes in recent years, with sustainability emerging as a crucial determinant of destination competitiveness (1). Accommodation establishments, as foundational elements of tourism infrastructure, play a pivotal role in converting sustainability commitments into enduring value for destinations (2). This is particularly critical for emerging tourism markets in Central Asia, where rapid growth necessitates development strategies that harmonize economic, social, and environmental priorities within a coherent policy framework (3).

Uzbekistan epitomizes this dynamism. By 2025, international visitor arrivals totaled 10.7 million, reflecting a 73% rise from pre-pandemic 2019 levels, propelled by nearly USD 6.5 billion in infrastructure investment over eight years (4). This transformation positions Uzbekistan as a compelling case for analyzing how sustainability-driven policies can enhance accommodation sector competitiveness. Recent research has further highlighted the significance of strategic communication in tourism marketing for improving destination recognition and differentiation (5–7).

This opinion article examines the relationship between sustainability practices, event tourism development, and accommodation competitiveness in emerging Central Asian destinations. We draw explicitly on three established theoretical foundations: Porter's (8) competitive advantage framework at the enterprise level; the Ritchie and Crouch (9) destination competitiveness model, which identifies core and supporting determinants including infrastructure and quality management; and Elkington's (10) Triple Bottom Line (TBL) framework, which delineates the economic, environmental, and social pillars of sustainable development. The Dwyer and Kim (11) integrated model further informs our analysis by emphasizing endowment factors alongside situational conditions and demand parameters. Drawing on these frameworks, we propose a refined conceptual model and derive practical implications for accommodation managers and destination policymakers.

## Sustainability and accommodation competitiveness: a theoretical foundation

The Ritchie and Crouch (9) destination competitiveness model identifies a hierarchy of determinants ranging from core resources and attractors to supporting factors and resources, policy and planning, and qualifying determinants. Central to this hierarchy—and echoed in the Dwyer and Kim (11) model—is the recognition that tourism infrastructure and quality management systems are indispensable supporting conditions without which even the richest endowments fail to generate competitive advantage. Elkington's (10) TBL reinforces this by insisting that sustainable competitiveness must be evaluated simultaneously across economic, environmental, and social dimensions, none of which can be treated in isolation.

Uzbekistan's accommodation sector has grown substantially, expanding from approximately 20,000 hotel rooms in 2018 to 6,861 properties with 184,000 beds by early 2025. International operators such as InterContinental and Accor have entered the market, applying global operational standards, sustainability protocols, and quality certification systems (12). Tourism revenue increased from USD 1.61 billion in 2022 to USD 3.52 billion in 2024, with the Uzbekistan-2030 Strategy targeting annual revenues of USD 5–6 billion (4). Average visitor length of stay has grown from 3–4 days to 7–9 days, signaling effective product diversification and improvements in accommodation quality standards.

Multiple pathways connect sustainability to accommodation competitiveness, each corresponding to a dimension of the TBL. From an economic dimension, eco-efficiency measures such as energy management systems and water conservation generate cost savings estimated at 15%–30% of operating expenses, improving profit margins and enabling competitive pricing (13). Environmentally, green certifications such as Green Key and EarthCheck provide differentiation potential, enabling certified properties to command premium pricing and enhance brand perception among environmentally conscious travellers (14, 15). Socially, local sourcing and community engagement initiatives foster authentic guest experiences and secure the social licence to operate, aligning with the Resource-Based View (16) wherein cultural authenticity serves as a unique competitive asset. Uzbekistan's four UNESCO World Heritage cities provide a significant heritage endowment that substantiates the Ritchie and Crouch (9) model's assertion that cultural and natural resources are core attractors underlying long-term competitiveness.

## Infrastructure as a foundational competitiveness determinant

Both the Ritchie and Crouch (9) and Dwyer and Kim (11) frameworks explicitly classify infrastructure as a critical supporting determinant of destination competitiveness. Infrastructure encompasses not only physical transport connectivity and accommodation capacity but also ICT systems, utility networks, and service accessibility for diverse visitor segments. Uzbekistan's USD 6.5 billion investment programme over the past eight years has yielded substantial advances in road and rail connectivity, airport modernisation, and the development of purpose-built tourism and conference complexes such as the Silk Road Samarkand precinct. However, infrastructure development must be sustainable in both design and operation if it is to deliver TBL outcomes. Energy-efficient building standards, water management systems, and accessible design requirements should be embedded as non-negotiable specifications in all new accommodation infrastructure to ensure alignment with Elkington's (10) economic, environmental, and social imperatives simultaneously.

## Quality standards as a driver of competitive positioning

Quality standards represent another determinant explicitly foregrounded in the Ritchie and Crouch (9) and Dwyer and Kim (11) models but frequently underemphasised in practice-oriented discussions of accommodation competitiveness. Consistent quality assurance systems—whether expressed through international classification schemes, ISO certification, or sector-specific hospitality standards—signal reliability to prospective visitors and business travellers, thereby supporting premium positioning and repeat visitation. Managerial attitudes toward environmental management within the accommodation sector are a critical mediating factor here: where managers lack awareness of or commitment to sustainability standards, the adoption of formal quality and certification systems remains limited (17). In the context of sustainability, quality standards increasingly intersect with green certification frameworks. Properties that hold internationally recognised green certifications such as GSTC Industry Criteria accreditation or Green Key status effectively communicate both service quality and environmental commitment to target audiences, generating competitive advantage across the economic, environmental, and social dimensions of the TBL. Uzbekistan's accommodation sector would benefit from a nationally coordinated quality assurance system that integrates sustainability performance indicators alongside traditional service quality metrics, creating a coherent signalling mechanism for both domestic and international markets.

## Event tourism as a competitiveness catalyst

Research on the sustainability legacies of mega-events (18) demonstrates that large-scale events can catalyse lasting improvements in sustainability performance across the accommodation sector. Properties that develop and implement comprehensive sustainability management systems in preparation for major events frequently acquire organisational competencies that yield efficiencies well beyond the event lifecycle (19). This concept of ‘sustainability-enabled resilience’ is especially pertinent to Uzbekistan, which has successfully hosted landmark international events including the SCO Summit (2022), the EBRD Annual Meeting (2023), and the 25th UNWTO General Assembly (2023). Each of these occasions has driven investment in accommodation infrastructure, quality service standards, and sustainability practices, generating legacy benefits consistent with both the Ritchie and Crouch (9) model's emphasis on event-driven competitiveness enhancement and the TBL's requirement for balanced economic, social, and environmental outcomes.

MICE (Meetings, Incentives, Conferences, Exhibitions) tourism has emerged as a strategic priority, accounting for an estimated 15%–18% of total tourism revenue in 2024, up from 10% in 2019 (20). Business travellers spend approximately USD 1,300 per trip compared with USD 750 for leisure visitors, and their demand patterns help mitigate seasonal fluctuations that undermine financial sustainability for accommodation operators (21). The Silk Road Samarkand complex, with its 28,000 m^2^ conference facility accommodating 3,500 delegates, exemplifies the strategic alignment of physical infrastructure investment with event tourism positioning. The upcoming 43rd UNESCO General Conference in Samarkand in 2025—the first held outside Paris in four decades—further demonstrates how sustained investment in sustainable infrastructure and quality standards can secure prestigious international events that in turn reinforce destination brand equity and accommodation competitiveness.

## Strategic communication, destination branding, and green certification awareness

The strategic communication of sustainability credentials and event-hosting capabilities is itself a vital competitiveness lever that operates through the mechanisms identified in the Ritchie and Crouch (9) and Dwyer and Kim (11) models. Radjabov et al. (6) argue that effective strategic communication in sustainable tourism promotion extends far beyond conventional marketing to encompass destination branding, stakeholder engagement, international positioning, and the dissemination of green certification credentials (7). These functions are not merely promotional; they are mechanisms through which the theoretical constructs of competitive advantage are operationalised in the marketplace.

Destination branding that foregrounds green certification is increasingly recognised as a driver of accommodation competitiveness across all three TBL dimensions. Economically, certified green properties achieve demonstrably higher occupancy rates and average daily rates in competitive markets (14, 15). Environmentally, the certification process itself drives measurable improvements in resource efficiency and waste reduction. Socially, transparent communication of sustainability credentials builds trust among local communities and international partners alike, reinforcing the social licence to operate that underpins long-term destination viability. For Uzbekistan, leveraging its UNESCO World Heritage positioning alongside internationally recognised green certification marks offers a distinctive branding proposition that is difficult for competitors to replicate, thereby providing the resource-based competitive advantage articulated by Barney (16) and operationalised within the Ritchie and Crouch (9) framework.

Green certification awareness must also be cultivated among accommodation operators themselves, particularly smaller and independent properties that may lack the managerial capacity or financial resources to pursue international certification independently. Destination management organisations and national tourism authorities play a critical facilitating role here, providing training, co-financing mechanisms, and marketing support so that the benefits of green certification are accessible across the full spectrum of the accommodation sector rather than confined to internationally affiliated chains.

## Inclusive tourism and the social sustainability dimension

The inclusive tourism dimension of competitiveness represents the social sustainability pillar of the TBL framework and is a determinant of long-term destination viability in both the Ritchie and Crouch (9) and Dwyer and Kim (11) models. Ibragimov et al. (22) demonstrate that competitive advantages deliver sustainable value only when they generate benefits for local communities and provide accessible experiences for diverse visitor demographics. Inclusive design in accommodation properties—currently implemented by only approximately 15% of properties in Uzbekistan—represents simultaneously a market growth opportunity and a social responsibility imperative. From a TBL perspective, inclusive tourism generates social returns through community employment, accessibility improvements, and equitable distribution of tourism revenues, while also contributing to economic sustainability through the diversification of the visitor base and the extension of the destination's accessible market.

## Discussion and refined conceptual framework

**Figure 1:**
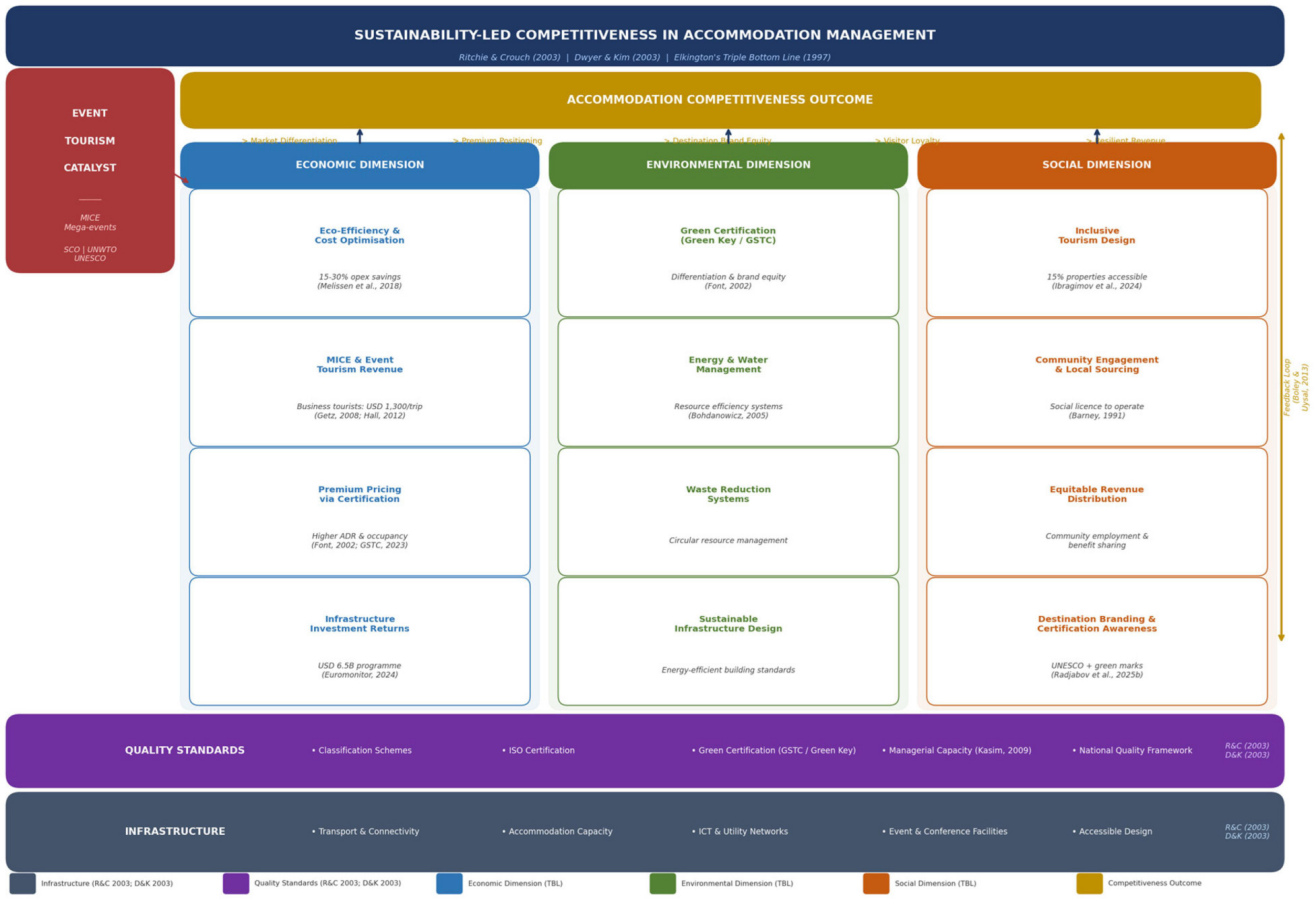
Conceptual framework: sustainability-led competitiveness in accommodation management (economic, environmental, and social dimensions). Source: Authors’ own elaboration, adapted from Ritchie and Crouch (9), Dwyer and Kim (11), and Elkington’s (10) triple bottom line framework.

Drawing on the preceding analysis and its grounding in the Ritchie and Crouch (9), Dwyer and Kim (11), and Elkington (10) frameworks, we present a refined conceptual framework (Figure 1) that integrates sustainability strategies, infrastructure development, quality standards, green certification branding, event tourism drivers, strategic communication, and inclusive tourism outcomes as interrelated determinants of accommodation competitiveness. The framework is structured around the TBL's three pillars—economic, environmental, and social—which serve as both the organising logic for individual strategies and the evaluation criteria for competitive outcomes.

The framework's economic dimension encompasses eco-efficiency and cost optimisation, MICE and event tourism revenue, premium pricing enabled by green certification and quality assurance, and infrastructure investment returns. The environmental dimension integrates green certification acquisition and communication, energy and water management, waste reduction, and sustainable infrastructure design standards. Sustainability methods—including energy efficiency, water conservation, waste management, and green certifications—directly improve operational performance and market positioning across both dimensions (23). The social dimension incorporates inclusive tourism design, community engagement, local sourcing, and equitable distribution of tourism economic benefits. Consistent with the Ritchie and Crouch (9) model, these three dimensions are supported by and embedded within a foundational infrastructure layer that conditions the capacity of the destination to deliver competitive accommodation experiences and by a quality standards layer that provides the operational framework through which sustainability commitments are translated into verifiable market signals.

The framework also acknowledges feedback loops through which competitive outcomes reinforce sustainability and event tourism investments via revenue generation, reputation enhancement, and stakeholder support (24). This dynamic relationship substantiates the thesis that sustainability-driven strategies generate virtuous cycles of competitive improvement rather than representing static trade-offs between profit and responsibility (1). Conceptualised as a dynamic system, Figure 1 illustrates how the determinants of accommodation competitiveness are not simply additive but mutually reinforcing: sustainable practices, event tourism development, and strategic destination branding operate in a recursive relationship in which each mechanism amplifies the effect of the others. Sustainability-led strategies are first operationalised through physical infrastructure and governance structures — Uzbekistan's USD 6.5 billion investment programme having established the foundational accommodation capacity and sustainable building standards required to host high-volume and high-value visitor flows. These infrastructure and governance commitments are then activated by event tourism demand: the successive hosting of the SCO Summit (2022), EBRD Annual Meeting (2023), and 25th UNWTO General Assembly (2023), together with the forthcoming 43rd UNESCO General Conference in Samarkand (2025), has converted capital investment into real market demand, driving occupancy, accelerating quality improvement cycles, and embedding sustainability management competencies across the accommodation sector that persist well beyond any individual event lifecycle (19). The competitive outcomes generated — occupancy growth, revenue uplift, and quality assurance advancement — are subsequently amplified through strategic communication that disseminates Uzbekistan's sustainability credentials, UNESCO World Heritage positioning, and event-hosting track record to international markets, reinforcing the brand signals that attract further investment mandates and premium visitor segments (6). Critically, these competitive outcomes feed back into further sustainability investment: revenue generated through premium positioning and MICE tourism provides the financial basis for green certification expansion, infrastructure enhancement, and inclusive tourism development, thereby sustaining the virtuous cycle and deepening the destination's competitive differentiation over successive planning horizons.

Framing accommodation as a central determinant of destination competitiveness — rather than a peripheral service component — has significant implications for both theory and the practical governance of Uzbekistan's tourism sector. While the Ritchie and Crouch (9) and Dwyer and Kim (11) models locate accommodation within the supporting resources and infrastructure category, the evidence from Uzbekistan demonstrates that accommodation is not merely supportive but constitutive of competitive positioning. The sector's expansion from approximately 20,000 hotel rooms in 2018 to 6,861 properties with 184,000 beds by early 2025, combined with the market entry of internationally branded operators applying global sustainability and quality standards, has repositioned the accommodation sector as an active generator of competitive advantage rather than a passive recipient of destination-level investment. This reframing is consistent with the Resource-Based View (16), wherein the accumulation of sustainability competencies, event management capabilities, and green certification credentials within the accommodation sector constitutes a portfolio of strategic assets that is both valuable and difficult for competing Central Asian destinations to replicate in the short term. Accordingly, accommodation development policy in Uzbekistan should be accorded equivalent strategic priority to destination marketing and physical infrastructure investment, with national quality and sustainability certification frameworks providing the institutional architecture through which individual property-level improvements aggregate into destination-level competitive advantage. The adoption of this approach is already discernible in the Uzbekistan-2030 Strategy's revenue targets and in the governance mandate of the Uzbekistan Tourism Development Agency, which coordinates sustainability standards, international event bidding, and destination branding within a single strategic framework — precisely the integrated policy architecture that the conceptual model in Figure 1 prescribes.

The framework's practical implications are explicitly grounded in its theoretical underpinnings. First, drawing on the economic dimension of the TBL and the Ritchie and Crouch (9) model's emphasis on policy and planning, sustainability expenditures should be positioned as competitiveness enablers rather than compliance costs, with clear communication of their integrated economic and environmental returns (13). Second, consistent with the infrastructure determinant foregrounded in both Ritchie and Crouch (9) and Dwyer and Kim (11), event tourism development must be anchored in sustainable infrastructure design to maximise legacy impacts and ensure that MICE investment generates enduring rather than ephemeral competitive advantage (19). Third, applying the branding and communication mechanisms identified in the Dwyer and Kim (11) model, the strategic communication of green certification credentials must be pursued collaboratively at both the property and destination levels to build a coherent and internationally legible market position (6). Fourth, operationalising the social dimension of the TBL, inclusive tourism development must be embedded in both accommodation design standards and destination management policy to ensure that competitive advantages generate broad societal benefits and sustain destination viability over the long term (25). Fifth, consistent with the quality standards determinant in both destination competitiveness models, a nationally coordinated quality and sustainability certification framework for Uzbekistan's accommodation sector would provide operators with a clear pathway to competitive differentiation and market recognition. Future research should empirically test this refined framework across diverse emerging destinations and develop quantitative indicators for sustainability–competitiveness linkages to validate the conceptual relationships proposed here.

## Conclusion

This opinion article has argued that sustainability-driven strategies, when systematically embedded within accommodation infrastructure, event tourism development, and strategic destination communication, function as dynamic accelerators of competitiveness rather than compliance-based constraints. The Uzbekistan case study substantiates a conceptualisation of accommodation competitiveness as a dynamic system of mutually reinforcing mechanisms in which each determinant — infrastructure and governance, sustainability performance, event tourism demand, and strategic communication — amplifies the competitive impact of the others, generating competitive advantage that is both cumulative, distinctive, and difficult for competing Central Asian destinations to imitate. The operationalisation of this system in Uzbekistan is evidenced by the convergence of USD 6.5 billion in infrastructure investment, successive high-profile event mandates, expanding green certification adoption, and the strategic communication of UNESCO World Heritage positioning in international markets, all of which feed back into further sustainability investment and destination brand strengthening in the manner illustrated by Figure 1.

Within this framework, competitiveness refers to the ability of accommodation establishments to: attract and retain tourism and event demand through differentiated product offerings, heritage positioning, and internationally recognised sustainability credentials; achieve financial performance and market differentiation by commanding premium pricing, sustaining high occupancy rates, and building durable brand equity across domestic and international market segments; and deliver superior value relative to competing destinations by integrating economic efficiency, environmental stewardship, and social inclusivity within a coherent operational model. These three dimensions of competitiveness are not independent objectives but are themselves mutually reinforcing: the delivery of superior comparative value reinforces brand differentiation, which in turn sustains the premium demand that finances further sustainability investment. The practical adoption of this framework in Uzbekistan requires a nationally coordinated policy architecture that embeds sustainability performance indicators within accommodation quality assurance standards, aligns event tourism infrastructure investment with sustainability legacy planning, and provides accessible pathways for smaller and independent properties to achieve green certification and participate in destination-level strategic communication. As Uzbekistan advances toward its Uzbekistan-2030 target of USD 5–6 billion in annual tourism revenues, the strategic imperative is clear: to position accommodation not as infrastructure background but as a primary vehicle through which sustainability commitments are converted into competitive outcomes, event tourism demand is captured and retained, and destination brand value is created and communicated to global markets.
